# When to Bleed or Not

**Published:** 1876-03

**Authors:** 


When We May Bleed and When We May Not Bleed.—In an address in the *British Medical Journal*, by Henry Moon, M.D., F.R.C.P., Physician to the Sussex County Hospital, the writer says:

In therapeutics there has been an immense improvement. I will only take one instance amongst many others, that of blood-letting. During my apprenticeship at a large London institution, my chief employment from day to day was to bleed and cup those patients who had been seen by the physicians and surgeons. Blood-letting was then used as a remedy whenever there was an increase of the temperature and a quickening of the pulse; and, doubtless, this indiscriminate irrational application of so bold a remedy destroyed thousands of lives annually. In some cases, however, blood-letting, with the light of modern science, is still a remedy of great practical usefulness.

We may bleed in, for instance, cerebral hemorrhage, if the impulse of the heart be strong, and its sounds loud; if the pulse be regular, and no signs of commencing œdema of the lungs exist, we should bleed without delay. Here a judicious timely bleeding may prevent the extension of the paralysis from the cerebrum to the medulla oblongata, which is essential to life.

In order that as much arterial blood as possible may enter the brain, we must try to facilitate the escape of venous blood, without, however, diminishing the propelling powers too much.

We may not bleed when, on the contrary, the heart's impulse is weak, the pulse irregular, and rattling in the trachea has already begun ; we may be almost certain bleeding will do harm, since the action of the heart, which is already weakened, would be still more impaired, and the amount of arterial blood going to the brain would be thus still more decreased. When these conditions occur, the indications requre just the opposite treatment, in spite of the original disease being the same, and being due to the same causes. . Here, by the use of stimulants, we must strive to prevent paralysis of the heart ; frictions, sinapisms, wine, ether, and musk, instead of bleeding, are called for.

We may bleed in acute croupous pneumonia, when the pneumonia has attacked a vigorous and hitherto healthy person, and is of recent occurrence, the temperature being higher than 105 deg. Fahr., and the pulse rating at more than 120 beats in a minute. Here danger only threatens from the violence of the fever ; and a free venesection will reduce the temperature, and lessen the frequency of the pulse. In those, however, who are already debilitated, bleeding increases the dangers of exhaustion. Should the fever in pneumonia be moderate, blood-letting is not indicated, even in healthy and vigorous individuals. It cannot cut the fever short ; indeed, the fever is more apt to persist, although in a somewhat more moderate degree ; so that the enfeebled patient is thrown into greater danger than if he had to pass through a more violent fever, but with unreduced strength.

We may bleed in fluxion of the lung, arising from excessive cardiac action threatening life. The result of a blood venesection here is astonishing; as soon as the volume of blood has become lessened, the pressure diminishes in the arteries (as it depends upon two forces: first, the energy of the cardiac contractions; secondly, the fullness of the cavities of the heart). The patients often breathe more freely during the operation, the bloody foam which they were expectorating vanishes, and life may be rescued from the greatest dangers, by aid of the physician.

So, also, in collateral fluxion of the lung (acute hyperæmia) during the course of acute pneumonia, pleurisy, or pneumothorax, we may bleed. Here a large part of the dyspnœa depends upon the overfilling of the capillaries and swelling of the vesicles in the portions of the lung unaffected by the inflammation.

When patients die in the first stages of pneumonia or pleurisy, or shortly after the air has penetrated into the pleural sac and compressed the lung (pneumothorax), they die of collateral fluxion (hyperæmia) and collateral œdema. If collateral fluxion threatens life during the progress of these diseases; if the patient be attacked with intense dyspnœa, and a moist *rale* become audible; if the sputa become serous, the danger is imminent. Then pay no regard to the small pulse, or rather, look upon it as a new reason for bleeding; for thereby the force of the heart is diminished, the pressure in the arteries of the hyperæmic parts of the lungs is also reduced; the capillaries are less full; the transudation of serum, which was threatening, or had already set in, does not occur or ceases; and here, too, we often see the patient breathe more freely and deeply when the blood is flowing.

Since, however, in by far the greater number of cases venesection has an unfavorable effect upon the main disease, by increasing the danger from exhaustion and impoverishment of the blood, we should not be led astray by these striking instantaneous results, so as to bleed without necessity; that is to say, unless life be threatened. Should œdema threaten in the course of disease of the heart, immediate danger to life may demand a diminution of the volume of the blood, and the relief consequent on venesection is usually beneficial.

The blood of persons of long standing disease of the heart is poor in fibrin and albumen, and has great tendency to form serous transudations. Venesection renders it thinner, and, therefore, bleeding should never be used in these cases but under the most pressing necessity.

Blood-letting should never be used in the hyperæmia of asthenic fever, no matter how great, and though the œdema threaten life.

In endocarditis, as a rule, bleeding is decidedly injurious ; still a condition sometimes exists where the indication as to symptoms calls for venesection. In cases where overcharge of the pulmonary circulation imperils life by threatening œdema of the lungs, and demands prompt relief by diminution of the volume of the blood. A great acceleration of the pulse, and signs of feebleness, however, in the action of the heart, indicates the administration of digitalis. Should palsy of the heart threaten, give stimulants boldly.—*Medical and Surgical Reporter.*

